# Diagnostic utility of IL-18 plasma levels in distinguishing abdominal from non-abdominal sepsis

**DOI:** 10.3389/fimmu.2025.1591262

**Published:** 2025-05-29

**Authors:** Anna Herminghaus, Mykola Totskyi, Christian Vollmer, Thomas Dimski, Timo Brandenburger, Anne Kuebart, Helen Rinderknecht, Christian B. Bergmann, Ina Vernikouskaya, Jasmin Maria Bülow, Nils Becker, Borna Relja

**Affiliations:** ^1^ Department of Anesthesiology, University Hospital Duesseldorf, Duesseldorf, Germany; ^2^ Department of Anesthesia and Intensive Care Medicine, Acibadem Tashkent International Medical Center, Tashkent, Uzbekistan; ^3^ Department of Trauma, Hand, Plastic and Reconstructive Surgery, Translational and Experimental Trauma Research, Ulm University Medical Center, Ulm, Germany; ^4^ Department of Internal Medicine II, Ulm University Medical Center, Ulm, Germany

**Keywords:** prediction, inflammation, cytokines, outcome, IL-18

## Abstract

**Background:**

Abdominal sepsis is a critical and high-risk condition in intensive care, characterized by diagnostic challenges, complex treatment, and high mortality. Non-specific symptoms and the difficulty of discriminating harmful bacteria from the normal flora complicate a timely diagnosis and treatment. Although timely interventions are crucial, the best timing of surgery remains uncertain, especially in unstable patients. Diagnostic markers like C-reactive protein, procalcitonin, and interleukins help guide diagnosis but often lack specificity of an abdominal focus. This study aims to identify possible additional markers for earlier detection of abdominal sepsis.

**Methods:**

Plasma samples were collected from 47 sepsis patients at the day of sepsis diagnosis, and from 10 healthy controls. Patients were retrospectively categorized into those with abdominal (n=23) and those with non-abdominal (n=24) sepsis. Patient`s characteristics, clinical outcomes, physiological and laboratory parameters, and cytokine levels were assessed. Receiver operating characteristic curves and Spearman correlation analyses were conducted.

**Results:**

Age and sex proportions were comparable across the sepsis groups, as were the chronic disease prevalence, the severity of illness and mortality rates. Patients with abdominal sepsis were more likely to undergo emergency surgeries. Pro-inflammatory cytokines like IL-6, MCP-1, and IL-18 were elevated, as was the anti-inflammatory IL-10 in both sepsis cohorts compared to healthy controls. IL-18 was particularly associated with a more severe inflammatory response in non-abdominal sepsis. IL-18 levels below 1892.00 pg/mL showed 82.6% sensitivity and 56.5% specificity for identifying patients with abdominal sepsis, with a significant diagnostic accuracy (AUC 0.68, p = 0.034). This suggests IL-18 as a useful additional moderate predictor for critical cases.

**Conclusion:**

The results demonstrate that IL-18, IL-6, MCP-1, and IL-10 are increased in sepsis, while IL-18 may serve as an additional biomarker for distinguishing abdominal from non-abdominal sepsis.

## 1 Introduction

Despite continuous progress in intensive care medicine, sepsis remains a major cause of morbidity and mortality in the intensive care unit (ICU), with outcomes strongly influenced by the timelines and appropriateness of early management (1). One of the most critical early steps in managing sepsis is identifying the source of infection, which directly informs decisions regarding antimicrobial therapy and the need for surgical or procedural source control. In particular, distinguishing between abdominal and non-abdominal sources is essential, as intra-abdominal infections frequently necessitate urgent interventions, whereas non-abdominal infections may follow a different clinical trajectory.

Abdominal sepsis is a specific and particularly severe form of sepsis, often caused by intra-abdominal abnormalities such as gastrointestinal perforation, ischemic bowel, or cholecystitis; associated with a high mortality rate of nearly 30% (2). These infections are clinically challenging due to varied manifestations and frequently require complex therapeutic regimes including antibiotic treatment and surgical source control. They can involve a broad spectrum of pathogens, including Gram-positive and Gram-negative bacteria, fungi, and parasites, necessitating microbiological diagnostics that distinguish commensal flora from potential pathogens (3, 4). In contrast, non-abdominal infections like pneumonia, urinary tract infections, or catheter-related bloodstream infections typically demand a different therapeutic approach. In a large cohort, Reitz et al. showed that initiating source control within six hours of recognition reduced the adjusted odds of 90-day mortality by 29% compared to delayed intervention (5). These findings align with earlier studies showing that inappropriate antimicrobial therapy and delayed source control significantly increase mortality in sepsis, particularly in abdominal cases (6–8). Surgical source control, although critical, is complicated by the patient`s hemodynamic stability and the risk associated with urgent surgery (7). Failure to identify and manage intra-abdominal sources in a timely manner is associated with increased morbidity and mortality (9). For instance, the multicenter observational study (AbSeS) found that emergency surgery within 2 hours of peritonitis diagnosis was associated with higher mortality compared to urgent intervention within 2–6 hours after diagnosis (7), whereas another study revealed a 100% mortality when surgical source control was delayed beyond 6 hours after gastrointestinal perforation (5, 6, 10). Conversely, Martinez et al. found no clear survival benefit from early (<12 hours) versus late intervention (11).

Effective source control is also needed to avoid the development of antimicrobial resistance. Lack of source control often implies prolonged antimicrobial therapy, which is a key driver of antimicrobial resistance (12). Notably, the empiric antibiotic regimen in abdominal sepsis often differs from that used in non-abdominal sepsis. Intra-abdominal infections tend to be polymicrobial and may include anaerobes and resistant Gram-negative organisms, requiring broader initial antimicrobial coverage (3, 7, 9). In contrast, non-abdominal infections like pneumonia or urinary tract infections may require a different, narrow spectrum antibiotic empiric strategy. Misguided therapy can lead to prolonged courses of antibiotics, increasing the risk for antimicrobial resistance and complications like *Clostridioides difficile* infection (13). The increasing prevalence of extended-spectrum β-lactamase (ESBL)-producing *Enterobacteriaceae* in intra-abdominal infections underscores the importance of timely, targeted therapy based on early source identification (14).

Prognostically, abdominal sepsis carries a significant burden. A large-scale cohort analysis reported an in-hospital mortality rate of 18.93% for abdominal sepsis—comparable to pulmonary sources (19.27%) but higher than renal (12.81%) or catheter-related infections (12), underscoring the prognostic importance of early source differentiation. This stratification can help clinicians prioritize risk assessment, prognostication, and resource allocation—such as ICU bed triage or surgical prioritization. Moreover, up to 31% of patients undergoing emergency surgery for peritonitis had undiagnosed sepsis prior to focused screening using SOFA scores, highlighting persistent diagnostic delays in abdominal sepsis (15). These delays can compromise resuscitation and preoperative optimization, especially in resource-limited settings.

Understanding the peritoneal immune response and the pathophysiology of abdominal sepsis is key to improving clinical outcomes. Early identification of the infection source is critical, yet intra-abdominal infections often present with a broad spectrum of symptoms, ranging from mild discomfort in localized abscesses to rapid progression to septic shock in cases of gastrointestinal perforation (16). Tools like the Mannheim Peritonitis Index may help identify high-risk patients, while early recognition, fluid resuscitation, appropriate antibiotic therapy, and prompt surgical intervention remain foundational elements of management (5–7, 17). Laboratory markers such as white blood count, C-reactive protein (CRP), and procalcitonin (PCT) are commonly used, but each has limitations. CRP and PCT may rise during bacterial infections, yet lack specificity and can be affected by trauma or surgery (16). Serum lactate indicates tissue hypoperfusion but is not specific for abdominal causes (18). Interleukin (IL)-6, a standard marker in many ICUs, correlates with an poor outcomes in sepsis but does not differentiate infection sources (19). Recently, IL-18, a proinflammatory cytokine of the IL-1 family, has gained attention as a potential biomarker in septic states (20, 21). It plays a role in innate immune responses and mucosal inflammation (22–24). However, its relevance in abdominal sepsis remains unclear. Some data suggest that IL-18 may help differentiate sepsis origins, offering a path toward source-specific diagnosis (20, 21).

Given these challenges, improved diagnostic tools are needed. This study aimed to evaluate whether IL-18 levels differ between abdominal and non-abdominal sepsis, and assess its utility as an early diagnostic indicator in critically ill patients.

## 2 Material and methods

### 2.1 Ethics

All patients included in the study were treated in the ICU at the University Hospital of the University Duesseldorf. The institutional ethics committee approved the study under the number: 2018094832. The healthy subjects were included at the University Hospital of the University Ulm upon the approval of the study by the institutional ethics committee (number: 420/23). The study was performed in accordance with the Declaration of Helsinki and following STROBE-guidelines. In alignment with the ethical standards, written informed consent was obtained from all enrolled subjects, and all enrolled subjects signed the informed consent forms themselves or informed consent was obtained from the nominated legally authorized representative, who consented on the behalf of participants as approved by the ethical committees. This study was conducted as a mixed retrospective, non-interventional observational study.

### 2.2 Study design and patient classification

This was a mixed retrospective observational study involving critically ill patients with sepsis admitted to the ICU. For blood sampling, patients were prospectively enrolled; however, the cytokine assessments were conducted retrospectively. The study population consisted of patients who were retrospectively classified into two subgroups based on the presumed or microbiologically confirmed infection source: Abdominal sepsis was defined as sepsis with an intra-abdominal focus, including but not limited to gastrointestinal or gastric perforation, anastomotic leakage, appendicitis, intestinal ischemia, or cholecystitis. Non-abdominal sepsis included all other sepsis etiologies such as pneumonia, urosepsis, and empyema, in the absence of confirmed abdominal pathology.

The diagnosis of sepsis in all patients was based on the Sepsis-3 criteria (25), which includes suspected or confirmed infection plus an increase in SOFA score ≥ 2 from baseline.

### 2.3 Inclusion and exclusion criteria

A total of forty-seven patients between 18 and 80 years of age were included. The study exclusion criteria were: age under 18 years, lack of or withdrawal of informed consent, refusal to participate in the study, expected ICU discharge within 72 hours, palliative care patients or those with established therapeutic limitations, foreseeable and unavoidable death at the time of screening, readmission after prior study inclusion (no repeat enrollment). Ten sex-matched healthy subjects (3 female and 7 male) with a mean age of 37.5 from the volunteering hospital staff were included as controls for cytokine analysis.

### 2.4 Initial patient assessment, treatment and clinical data acquisition

Patients were managed according to international sepsis guidelines (13). Clinical data including the Sequential Organ Failure Assessment Score (SOFA) score, Acute Physiology And Chronic Health Evaluation (APACHE) II, blood pressure, heart and respiratory rate, body temperature, routine blood gas analysis (including pH and lactate levels), glucose, as well as coagulation parameters (thromboplastin time, TPT; partial TPT, PTT; international normalized ratio, INR; fibrinogen, and platelets, PLT) were recorded at ICU admission and on the day of sepsis diagnosis (d0), and tracked up to 7 days post- diagnosis. Functional organ parameters including creatinine, bilirubin, glutamate dehydrogenase (GLDH), gamma-glutamyl transferase (GGT), creatine kinase (CK), and the numbers of transfused packed red blood cell (PRBC) units and fresh frozen plasma (FFP) were recorded. Furthermore, C-reactive protein (CRP) and procalcitonin (PCT) were determined.

Additional diagnostic imaging (X-rays, CT scans, MRI, bronchoscopy, endoscopy, etc.) was performed as clinically indicated. Information on emergency surgical interventions and infection source was recorded for classification and comparative analysis. The length of hospital stay before ICU admission, ICU length of stay, and in-hospital mortality were also documented.

### 2.5 Blood processing and analyses

Blood samples were obtained for clinical routine diagnostic parameters at ICU admission (ICU adm.) and at the day of sepsis diagnosis (d0) in citrate and ethylenediaminetetraacetic acid (EDTA) tubes (BD vacutainer, Becton Dickinson Diagnostics, Aalst, Belgium). Plasma for cytokine analyses was obtained from EDTA tubes at d0 processed via centrifugation at 2000 × g for 15 minutes at 4°C. Samples were stored at -80°C until analysis. The same procedure was applied for healthy controls.

### 2.6 Analysis of circulating cytokines and chemokines

Thirteen inflammatory mediators including IL-1β, IFN-α2, IFN-γ, TNF-α, MCP-1, IL-6, IL-8, IL-10, IL-12p (70), IL-17A, IL-18, IL-23 and IL-33 were measured using the commercially available LEGENDplex Human Inflammation Panel 1 kit (BioLegend, San Diego, California), according to manufacturer’s instructions. Samples were analyzed using the Attune CytPix flow cytometer (Thermo Fisher Scientific, Germany). Concentrations of each parameter were calculated based on standard curves and logarithmic transformation.

### 2.7 Statistical analysis

All data were tested for normality using the Kolmogorov-Smirnov test. To assess statistical differences between the groups, the unpaired non-parametric Mann Whitney U test was applied. Chi-square test was applied for the analyses of proportions. Correlation analysis was performed using Spearman’s correlation coefficient and Spearman’s r-test. Receiver–operator curves (ROC) were generated to analyze the optimal cutoff levels. Data are presented as the median ± interquartile range (IQR), mean ± standard error of the mean (sem) unless otherwise stated. A p-value < 0.05 was considered statistically significant. GraphPad Prism 10.0 software (GraphPad Software Inc. San Diego, CA) was used for statistical analyses.

## 3 Results

### 3.1 Patient characteristics and outcomes

The comparison between patients with abdominal sepsis and those with non-abdominal sepsis reveals that both groups had a similar number of patients (23 vs. 24), and the median age for abdominal sepsis patients was slightly higher (75 years vs. 67 years), though this difference was not statistically significant. Both groups had a similar proportion of female patients, around 30%, and the prevalence of chronic diseases was also comparable (26% in abdominal sepsis and 29% in non-abdominal), with no significant difference (**Table 1**).

**Table 1:** Overview of the characteristics of patients with abdominal and non-abdominal (non-abd.) sepsis.

Characteristics	Abdominal sepsis	Non-abd. sepsis	p <0.05
Number of patients	23	24	n.s.
Age (years) (IQR)	75.00 (61.00-81.00)	67.00 (55.25-74.75)	n.s.
Sex (female) (n, %)	7, 30.43%	7, 29.17%	n.s.
Chronic disease (n, %)	6, 26.09%	7, 29.17%	n.s.
Infection site			
Blood (n, %)	8, 34.78%	10, 41.67%	n.s.
Lung (n, %)	1, 4.35%	12, 50.00%	0.0007
Abdomen (n, %)	23, 100.00%	0, 0%	<0.0001
Other (n, %)	0, 0%	12, 50.00%	<0.0001
Surgical operation (n, %)	14, 60.87%	18, 75.00%	n.s.
Emergency surgical operation (n, %)	21, 91.30%	8, 33.33%	<0.0001
SOFA score ICU adm.	10.00 (7.50-12.00)	7.00 (5.00-11.00)	n.s.
SOFA score d0	9.00 (7.00-12.00)	9.00 (5.00-11.00)	n.s.
APACHE II ICU adm.	25.00 (16.75-29.75)	27.00 (19.00-31.00)	n.s.
APACHE II d0	27.00 (17.75-31.25)	25.00 (17.00-30.00)	n.s.
Length of hospital stay prior ICU (days)	0.00 (0.00-1.00)	8.50 (0.00-30.00)	0.0029
ICU days ≥7 (n, %)	16, 69.57%	15, 62.50%	n.s.
Length of ICU stay (days)	15.00 (4.00-25.00)	9.50 (4.00-22.75)	n.s.
Length of hospital stay (days)	28.00 (15.00-58.00)	39.50 (18.75-67.75)	n.s.
Mortality rate (n, %)	8, 34.78%	9, 37.5%	n.s.

A comparative analysis of demographic, clinical, and outcome parameters between patients diagnosed with abdominal and non-abdominal sepsis at the admission to the intensive care unit (ICU adm.) or at the day of sepsis diagnosis (d0) is demonstrated. Variables include the number of patients, age (median and interquartile range, IQR), sex distribution, presence of chronic disease, infection site (blood, lung, abdomen, or other), incidence of surgical intervention and emergency surgery, severity scores (Sequential Organ Failure Assessment Score, SOFA and Acute Physiology And Chronic Health Evaluation, APACHE II), length of hospital stay before ICU admission, duration of ICU stay, total hospital stay, and mortality rate. Data are given as median and interquartile range (IQR) with 25% and 75% percentile. Statistical comparisons between groups are indicated, with p-values less than 0.05 denoting significant differences. Non-significant results are labeled as “n.s.”.

The infection sites differed markedly between the two groups. In the abdominal sepsis group, in 100% of patients, abdominal infections were confirmed, while none of the patients in the non-abdominal sepsis group had infections in this site. Conversely, 50% of the non-abdominal sepsis group had lung infections, compared to 4% in the abdominal sepsis group (p = 0.0007, **Table 1**). Additionally, 50% of the non-abdominal sepsis patients had infections in other sites, while no patients in the abdominal sepsis group had infections outside the abdomen (p < 0.0001, **Table 1**). Blood infections were observed at similar rates in both groups (35% in abdominal sepsis and 42% in non-abdominal sepsis), with no significant difference (**Table 1**).

The overall rate of performed surgery was not significantly different between the two groups. However, emergency surgeries were more common in abdominal sepsis patients, with 91% undergoing emergency operations compared to 33% in the non-abdominal sepsis group (p < 0.0001, **Table 1**).

Regarding disease severity, there were no significant differences in the SOFA and APACHE II scores at ICU admission or on d0, indicating that both groups had similar severity of illness at these points (**Table 1**).

Length of hospital stay before ICU admission was significantly shorter for abdominal sepsis patients, with a median of 0 days compared to 8.5 days in the non-abdominal sepsis group (p = 0.0029, **Table 1**). However, there were no significant differences between the groups in terms of total ICU stay, total hospital stay, or the proportion of patients staying in the ICU for 7 days or more (**Table 1**).

Finally, the hospital mortality rates were similar between the two groups, with 34.78% mortality in the abdominal sepsis group and 37.5% in the non-abdominal sepsis group, showing no significant difference in outcomes (**Table 1**).

In summary, patients with abdominal sepsis were more likely to have emergency surgeries, had distinct infection sites, and shorter hospital stays prior to ICU admission compared to those with non-abdominal sepsis. However, the overall severity of illness and mortality were similar between the two groups.

**Figure 1:**
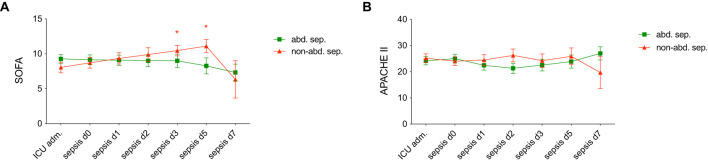
Comparison of **(A)** Sequential Organ Failure Assessment (SOFA) scores and **(B)** Acute Physiology and Chronic Health Evaluation II (APACHE II) scores in patients with abdominal sepsis (abd. sep.) and non-abdominal (non-abd. sep.) sepsis at various time points: admission to the intensive care unit (ICU adm.), on day of the sepsis diagnosis (d0), and on days 1, 2, 3, 5, and 7 post-sepsis onset. Each data point represents the mean score, with error bars indicating standard error of the mean. Statistically significant differences are marked as *p <0.05 vs. corresponding matched-pair at the ICU adm.

**Figure 1** displays a comparison between abdominal and non-abdominal sepsis patients over time in terms of several clinical severity scores: SOFA and APACHE II. **Figure 1A** shows a significant increase in SOFA scores in non-abdominal sepsis group over time, with notable statistical differences at d3 and d5 (p < 0.05). The APACHE II score reflecting the overall severity of illness, shows high scores at ICU admission in both groups. However, there are no significant differences between the two groups in this parameter (**Figure 1B**).

### 3.2 Physiological and laboratory parameters

The comparison of physiological and laboratory parameters between patients with abdominal sepsis and those with non-abdominal sepsis reveals several notable findings. In terms of blood pressure and heart rate, there were no significant differences between the two groups. Systolic blood pressure (SBP), shock index, and heart rate at ICU admission, on d0 (day of sepsis diagnosis) were similar in both groups (**Table 2**). Mean arterial blood pressure values followed a similar trend, showing no significant differences between abdominal and non-abdominal sepsis patients at any of the measured time points (**Table 2**).

**Table 2:** An overview of the physiological characteristics and laboratory parameters of the study population with abdominal and non-abdominal (non-abd.) sepsis at the admission to the intensive care unit (ICU adm.) and at the day of sepsis diagnosis (d0) is shown.

Physiological and laboratory parameters	Abdominal sepsis	Non-abd. sepsis	p <0.05
SBP (mm Hg) ICU adm.	107.40 ± 21.58	99.17 ± 22.05	n.s.
SBP (mm Hg) d0	99.77 ± 15.92	96.67 ± 15.01	n.s.
Shock index (HR / SBP) ICU adm.	1.002 ± 0.381	1.163 ± 0.572	n.s.
Shock index (HR / SBP) d0	0.975 ± 0.246	0.988 ± 0.365	n.s.
Heart rate ICU adm.	100.40 ± 25.49	107.50 ± 29.27	n.s.
Heart rate d0	97.50 ± 20.16	93.75 ± 24.51	n.s.
MABP (mm Hg) ICU adm.	71.48 ± 13.61	65.58 ± 13.44	n.s.
MABP (mm Hg) d0	67.77 ± 9.22	64.42 ± 9.17	n.s.
FiO_2_ (%) ICU adm.	55.74 ± 19.03	47.39 ± 27.64	n.s.
FiO_2_ (%) d0	42.59 ± 16.17	42.39 ± 24.68	n.s.
PO_2_ (%) ICU adm.	114.30 ± 57.10	94.88 ± 45.04	n.s.
PO_2_ (%) d0	89.95 ± 23.58	86.65 ± 27.58	n.s.
PCO_2_ (%) ICU adm.	42.83 ± 11.28	39.83 ± 12.28	n.s.
PCO_2_ (%) d0	44.77 ± 9.72	43.13 ± 11.79	n.s.
Horowitz index ICU adm.	245.60 ± 116.60	303.60 ± 226.20	n.s.
Horowitz index d0	254.50 ± 81.04	262.30 ± 140.30	n.s.
PEEP (cm H_2_O) ICU adm.	6.95 ± 3.68	4.52 ± 4.86	0.0494
PEEP (cm H_2_O) d0	7.30 ± 2.90	6.10 ± 6.10	n.s.
Hemoglobin (g / dL) ICU adm.	11.17 ± 2.59	9.88 ± 1.25	n.s.
Hemoglobin (g / dL) d0	10.42 ± 2.00	9.07 ± 1.26	0.0134
Hematocrit (%) ICU adm.	35.73 ± 8.74	30.92 ± 3.18	n.s.
Hematocrit (%) d0	33.65 ± 7.20	29.66 ± 4.08	0.0255
Glucose (mg / dL) ICU adm.	136.90 ± 55.16	147.50 ± 55.81	n.s.
Glucose (mg / dL) d0	131.00 ± 33.16	128.20 ± 38.01	n.s.
Lactate (g / dL) ICU adm.	4.88 ± 4.12	3.23 ± 2.98	n.s.
Lactate (g / dL) d0	4.28 ± 3.59	3.37 ± 4.27	n.s.
HCO_3_ ICU adm.	22.49 ± 4.73	22.58 ± 3.16	n.s.
HCO_3_ d0	25.83 ± 4.75	24.93 ± 3.68	n.s.
Base excess ICU adm.	-2.109 ± 5.436	-2.200 ± 3.868	n.s.
Base excess d0	1.536 ± 5.656	0.104 ± 4.26	n.s.
PH value ICU adm.	7.347 ± 0.081	7.390 ± 0.101	n.s.
PH value d0	7.375 ± 0.091	7.402 ± 0.069	n.s.
TPT (%) ICU adm.	24.00 ± 9.16	40.79 ± 44.36	n.s.
TPT (%) d0	30.95 ± 31.83	37.91 ± 44.91	n.s.
INR (sec.) ICU adm.	1.277 ± 0.271	1.642 ± 0.949	n.s.
INR (sec.) d0	1.365 ± 0.313	1.567 ± 0.603	n.s.
PTT (sec.) ICU adm.	30.22 ± 9.11	38.58 ± 16.18	n.s.
PTT (sec.) d0	34.59 ± 17.96	38.04 ± 11.17	n.s.
TT (sec.) ICU adm.	28.26 ± 27.49	39.65 ± 42.88	n.s.
TT (sec.) d0	30.39 ± 30.44	38.64 ± 44.84	n.s.
Fibrinogen (mg / dL) ICU adm.	433.10 ± 112.30	430.90 ± 221.50	n.s.
Fibrinogen (mg / dL) d0	433.60 ± 134.80	496.10 ± 228.60	n.s.
Leukocytes (U / nL) ICU adm.	13.95 ± 8.46	18.67 ± 10.44	n.s.
Leukocytes (U / nL) d0	13.74 ± 8.83	16.68 ± 9.45	n.s.
PLT count (U / nL) ICU adm.	258.40 ± 156.60	262.50 ± 145.20	n.s.
PLT count (U / nL) d0	251.40 ± 146.10	233.00 ± 136.60	n.s.
Erythrocytes (U / nL) ICU adm.	4.22 ± 2.41	3.32 ± 0.38	n.s.
Erythrocytes (U / nL) d0	3.97 ± 2.17	3.12 ± 0.36	0.0086
Creatinine (mg / dL) ICU adm.	1.499 ± 1.01	2.179 ± 2.531	n.s.
Creatinine (mg / dL) d0	1.414 ± 0.86	1.862 ± 1.841	n.s.
Bilirubin (mg / dL) ICU adm.	1.35 ± 1.37	1.33 ± 1.84	n.s.
Bilirubin (mg / dL) d0	1.37 ± 1.33	1.93 ± 2.56	n.s.
GLDH (U / L) ICU adm.	344.10 ± 247.40	381.20 ± 269.4	n.s.
GLDH (U / L) d0	352.50 ± 262.00	390.30 ± 239.40	n.s.
GGT (U / L) ICU adm.	82.85 ± 92.85	109.20 ± 82.24	n.s.
GGT (U / L) d0	66.26 ± 59.44	146.50 ± 147.00	0.0179
CK (U / L) ICU adm.	393.50 ± 755.00	216.70 ± 257.90	n.s.
CK (U / L) d0	507.20 ± 1083.00	217.50 ± 236.80	n.s.
Procalcitonin ICU adm.	21.88 ± 31.07	15.93 ± 27.50	n.s.
Procalcitonin d0	17.05 ± 24.41	32.99 ± 42.07	n.s.
CRP (mg / dL) ICU adm.	12.36 ± 12.83	15.34 ± 11.59	n.s.
CRP (mg / dL) d0	11.98 ± 10.42	17.23 ± 12.63	n.s.
Noradrenalin (mL / h) ICU adm.	14.09 ± 10.80	10.06 ± 10.48	n.s.
Noradrenalin (mL / h) d0	12.55 ± 10.27	11.38 ± 11.38	n.s.
PRBC transfusion (U) ICU adm.	0.17 ± 0.49	0.0 ± 0.0	n.s.
PRBC transfusion (U) d0	0.50 ± 0.80	0.42 ± 1.06	n.s.
FFP transfusion (U) ICU adm.	0.74 ± 1.10	0.50 ± 1.22	n.s.
FFP transfusion (U) d0	1.23 ± 1.60	0.83 ± 1.74	n.s.
Electrolytes (mL) ICU adm.	1519.00 ± 651.60	1693.00 ± 1167.00	n.s.
Electrolytes (mL) d0	2009.00 ± 1208.00	1946.00 ± 1179.00	n.s.

APACHE II, Acute Physiology and Chronic Health Evaluation II; CK, Creatine Kinase; CRP, C-Reactive Protein, FiO_2_, Fraction of Inspired Oxygen; FFP, Fresh Frozen Plasma; GGT Gamma-Glutamyl Transferase; GLDH, Glutamate Dehydrogenase; HCO_3_, Bicarbonate; HR Heart Rate; INR, International Normalized Ratio; MABP, Mean Arterial Blood Pressure; PCO_2_, Partial Pressure of Carbon Dioxide; PEEP, Positive End-Expiratory Pressure; PLT, Platelet; PO_2_, Partial Pressure of Oxygen; PRBC, Packed Red Blood Cells; SBP, Systolic Blood Pressure; SOFA, Sequential Organ Failure Assessment; TPT, Thrombin Time Percentage; TT, Thrombin Time; PTT, Partial Thromboplastin Time. Data are given as mean ± standard error of the mean, and significant differences between groups are indicated by p-values less than 0.05; non-significant results are marked as “n.s.”.

Regarding oxygenation and ventilation, no significant differences were observed in the fraction of inspired oxygen (FiO_2_) or partial pressure of oxygen (PO_2_) between the two groups at ICU admission, or on d0. However, partial pressure of carbon dioxide (PCO_2_) was marginally higher in abdominal sepsis patients at ICU admission (p = 0.0812, **Table 2**). A significant difference was found in positive end-expiratory pressure (PEEP), which was higher in abdominal sepsis patients at ICU admission (p = 0.0494), indicating more aggressive ventilatory support (**Table 2**).

For hemoglobin and hematocrit levels, abdominal sepsis patients had significantly higher hemoglobin on d0 (p = 0.0134, **Table 2**). Hematocrit levels were also significantly higher in this group on d0 (p = 0.0255, **Table 2**). Lactate levels were borderline higher in abdominal sepsis patients at ICU admission (p = 0.0540) and d0 (p = 0.0542), which could suggest higher levels of tissue hypoxia or metabolic stress, though these differences did not reach statistical significance (**Table 2**). Glucose levels remained similar between the two groups at all time points. In terms of acid-base balance and electrolytes, bicarbonate (HCO_3_) levels were not significantly different (**Table 2**). Additionally, pH values were marginally lower in abdominal sepsis patients at ICU admission (p = 0.0568), suggesting slight acidosis in this group (**Table 2**).

Clotting parameters showed no significant differences between the groups, indicating that coagulation function was comparable between the two groups at all time points (**Table 2**).

Regarding the inflammatory markers, both procalcitonin and CRP levels were similar between abdominal sepsis and non-abdominal sepsis patients, showing no significant differences in the inflammatory response (**Table 2**).

For liver function, there was one notable difference: GGT was significantly lower in abdominal sepsis patients on d0 (p = 0.0179, **Table 2**). However, other liver function markers, including bilirubin and GLDH levels were similar between the groups (**Table 2**).

Finally, there were no significant differences between the two groups in terms of red blood cell transfusions, fresh frozen plasma transfusions, or electrolyte volumes administered at any time point (**Table 2**).

### 3.3 Analyses of cytokines

**Figure 2:**
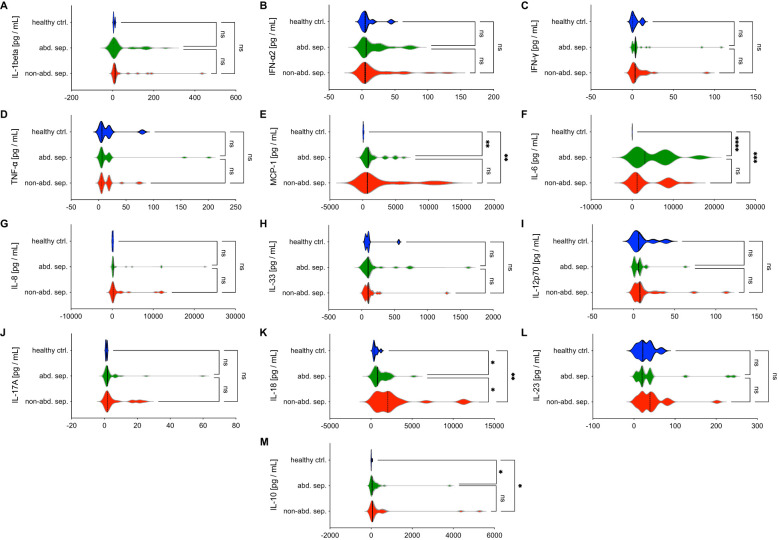
Comparative analysis of cytokine and chemokine levels in abdominal sepsis (abd. sep.) and non-abdominal sepsis (non-abd. sep.) patients versus healthy controls (ctrl). Panels **(A–M)** show the levels of various parameters, including **(A)** IL-1β, **(B)** IFN-α2, **(C)** IFN-γ, **(D)** TNF-α, **(E)** MCP-1, **(F)** IL-6, **(G)** IL-8, **(H)** IL-33, **(I)** IL-12p70, **(J)** IL-17A, **(K)** IL-18, **(L)** IL-23, and **(M)** IL-10 across the three groups. Cytokine concentrations are measured in picograms per milliliter (pg/mL) at the day of the sepsis diagnosis. Statistically significant differences are marked as *p <0.05, **p <0.01, ***p <0.001 and ****p <0.0001, while “ns” denotes non-significant comparisons.

The results in **Figure 2** show the levels of various cytokines in peripheral blood between patients with abdominal sepsis (abd. sep.), non-abdominal sepsis (non-abd. sep.), and healthy controls at the day of sepsis diagnosis (d0). The cytokines measured include pro-inflammatory markers such as IL-1beta, IL-6, IL-8, and TNF-α, among others.

IL-1beta, IFN-α2, IFN-γ, TNF-α, IL-8, IL-33, IL12p70, IL-17A, and IL-23 do not show significant differences between the sepsis groups or compared to healthy controls at d0, suggesting these markers may not play a central role in distinguishing between sepsis types (**Figure 2**).

MCP-1, IL-6, and IL-10 levels are significantly elevated at d0 in both sepsis groups compared to healthy controls (p < 0.05, **Figure 2**). MCP-1, which is responsible for recruiting monocytes to sites of infection, plays a vital role in the immune response to sepsis. Enhanced levels of IL-6, a key mediator of inflammation which is commonly associated with the acute phase of sepsis, suggest its critical role in driving the systemic inflammatory response.

IL-18 is significantly elevated in non-abdominal sepsis group compared to both abdominal sepsis group and healthy controls, highlighting a marked inflammatory response in this group (p < 0.05, **Figure 2**). IL-18 as a potent pro-inflammatory cytokine promotes the production of IFN-γ, which enhances the immune response. The elevated IL-18 levels in the non-abdominal sepsis group suggest its involvement in the more severe inflammatory response associated with another type of infection than abdominal causes.

### 3.4 Correlation analyses for patients with abdominal sepsis and clinical and experimental parameters

The correlation analysis for patients with sepsis reveals important relationships between certain clinical parameters and the stratification of sepsis (abdominal vs. non-abdominal sites). In this analysis, negative correlations indicate that an increase in a measured value is more strongly associated with abdominal sepsis. Conversely, positive correlations suggest that an increase in a parameter is more strongly linked to other forms of sepsis.

Additionally, when a decrease in a measured value is associated with abdominal sepsis, it is considered a positive correlation, meaning that lower values of the parameter are more common in abdominal sepsis patients. On the other hand, if lower values are linked to other types of sepsis, this is defined as a negative correlation. This approach helps to clarify which clinical parameters are more characteristic of either abdominal or other forms of sepsis based on whether values increase or decrease in association with each type.

Older age correlated negatively with non-abdominal sepsis (r = -0.369, p = 0.008, **Table 3**). In contrast, a higher BMI was positively correlated with non-abdominal sepsis (r = 0.443, p = 0.010, **Table 3**).

**Table 3:** Correlation analyses of physiological, laboratory, and clinical parameters in patients with abdominal and non-abdominal (non-abd.) sepsis at the admission to the intensive care unit (ICU adm.) or at the day of sepsis diagnosis (d0) showing results for abdominal sepsis.

Correlation analyses (abd. sep.)	Spearman r	p value
Age	-0.369	0.008
Sex	-0.297	0.034
BMI	0.443	0.010
RR diast. (mm Hg) ICU adm.	-0.361	0.009
RR diast. (mm Hg) d0	-0.377	0.007
Heart rate ICU adm.	0.287	0.043
Hypothermia (<36°C) ICU adm.	-0.333	0.017
Hypothermia (<36°C) d0	-0.420	0.002
PEEP (cm H_2_O) ICU adm.	-0.493	0.001
PEEP cm H_2_O) d0	-0.357	0.016
Hemoglobin ICU adm.	-0.395	0.005
Hemoglobin (g / dL) d0	-0.451	0.001
Glucose (mg / dL) ICU adm.	0.284	0.044
Lactate (g / dL) ICU adm.	-0.420	0.002
Lactate (g / dL) d0	-0.421	0.003
Base excess d0	-0.300	0.034
Acidosis (pH <7.35) ICU adm.	-0.327	0.019
Acidosis (pH <7.35) d0	-0.378	0.007
TPT (%) d0	0.304	0.040
Fibrinogen (mg / dL) d0	0.328	0.026
GLDH (U / L) ICU adm.	0.320	0.028
GGT (U / L) ICU adm.	0.347	0.006
GGT (U / L) d0	0.561	0.031
CRP (mg / dL) ICU adm.	0.395	0.004
CRP (mg / dL) d0	0.440	0.002
Hospital days before ICU	0.432	0.002
IL-18 (pg /m L) d0	0.316	0.032

The Spearman correlation coefficient (r) and p-values for various factors including demographic characteristics (age, sex, BMI), vital signs (diastolic blood pressure, heart rate, hypothermia status), respiratory support parameters, laboratory markers, and clinical are given. Abbreviations: BMI, Body Mass Index; CRP, C-Reactive Protein; GGT, Gamma-Glutamyl Transferase; GLDH, Glutamate Dehydrogenase; IL-18, Interleukin-18; PEEP, Positive End-Expiratory Pressure; RR diast., Diastolic Blood Pressure; TPT, Thrombin Time Percentage.

When examining blood pressure, higher diastolic readings at ICU admission (r = -0.361, p = 0.009), and on d0 (r = -0.377, p = 0.007) showed an association with abdominal sepsis, meaning lower diastolic blood pressure was linked to non-abdominal sepsis types (**Table 3**).

There was also a positive correlation between heart rate at ICU admission and outcomes (r = 0.287, p = 0.043), suggesting that higher heart rates were associated with non-abdominal sepsis types (**Table 3**).

Regarding ventilation and oxygenation, there were no correlations between FiO_2_ and PCO_2_ on d0 (**Table 3**). PEEP at ICU admission (r = -0.493, p = 0.001) and d0 (r = -0.357, p = 0.016) was negatively correlated, indicating that higher PEEP was associated with abdominal sepsis (**Table 3**).

The analysis showed significant correlations for hemoglobin levels at ICU admission (r = -0.395, p = 0.005), and d0 (r = -0.451, p = 0.001) with non-abdominal sepsis. Lower hemoglobin levels were linked to non-abdominal sepsis. Similarly, higher lactate levels at ICU admission (r = -0.420, p = 0.002) and d0 (r = -0.421, p = 0.003) were associated with abdominal sepsis (**Table 3**).

In terms of acid-base balance, lower base excess on d0 (r = -0.300, p = 0.034), as well as lower pH at ICU as well as on d0 correlated negatively with non-abdominal sepsis (**Table 3**).

Several clotting factors were positively correlated with the type of sepsis, including TPT (r = 0.304, p = 0.040) and fibrinogen (r = 0.328, p = 0.026) on d0, suggesting a link between clotting ability and as cause rather non-abdominal than abdominal sepsis site (**Table 3**).

Markers of organ injury as well as liver function showed significant positive correlations, with GLDH (r = 0.320, p = 0.028) at d0, and GGT at ICU admission (r = 0.347, p = 0.006) and d0 (r = 0.561, p = 0.031, **Table 3**).

C-reactive protein positively correlated with non-abdominal sepsis at ICU admission (r = 0.395, p = 0.004) and d0 (r = 0.440, p = 0.002).

Finally, a significant and positive correlation between IL-18 and non-abdominal sepsis is observed (r = 0.316, p = 0.032, **Table 3**).

### 3.5 Analyses and diagnostic performance of IL-18 for non-abdominal sepsis

**Table 4** shows a threshold of < 1892.00 pg/mL, with a sensitivity at 82.61% (CI 62.86-93.02) and a specificity of 56.52% (CI 36.81-74.37), with an AUC of 0.6825 (p = 0.0340), to predict abdominal sepsis (**Table 4**). This cut-off value was chosen based on the highest likelihood ratio for corresponding possible highest sensitivity and specificity values.

**Table 4:** Cut-off value of Interleukin-18 (IL-18) with corresponding sensitivity and specificity for distinguishing abdominal sepsis from non-abdominal sepsis at the day of sepsis diagnosis (d0).

Parameter	Value	Sensitivity %	95% CI	Specificity %	95% CI	Likelihood ratio	AUC	p value
**IL-18 d0**	<1892.00	82.61	62.86 to 93.02	56.52	36.81 to 74.37	1.900	0.6824	0.0340

AUC, Area Under the Curve; CI, Confidence Interval. A p-values less than 0 is considered significant.

**Figure 3:**
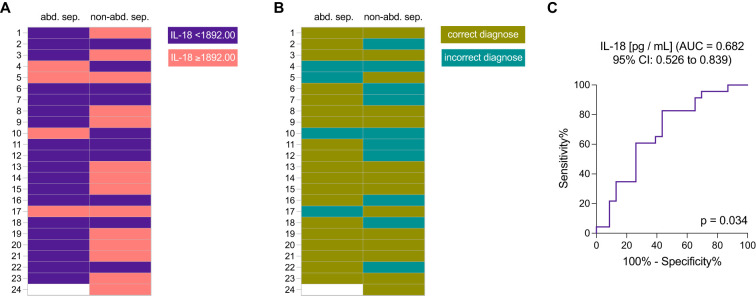
The diagnostic efficacy of interleukin-18 (IL-18) levels in distinguishing abdominal sepsis (abd. sep.) from non-abdominal sepsis (non-abd. sep.) patients. **(A)** Distribution of IL-18 levels (threshold of 1892.00 pg/mL) in patients with abdominal sepsis versus non-abdominal sepsis; and **(B)** diagnostic accuracy illustrated by correct and incorrect classifications based on IL-18 levels, with a threshold set at 1892.00 pg/mL. **(C)** Receiver Operating Characteristic (ROC) curve assessing the sensitivity and specificity of IL-18 as a biomarker for abdominal sepsis, showing the area under the curve (AUC).

**Figure 3** presents the diagnostic performance of IL-18 levels as a biomarker for distinguishing abdominal from non-abdominal sepsis cases. **Figure 3A** shows the real distribution of IL-18 concentrations across patient samples, highlighting a threshold at 1892.00 pg/mL, where IL-18 levels are categorized into two groups: values below and above the threshold. In the abdominal sepsis group 19 out of 23, and in the non-abdominal sepsis group 10 out of 23 patients had IL-18 levels below the cut-off (**Figure 3A**). **Figure 3B** illustrates the diagnostic accuracy with correct and incorrect classifications of abdominal sepsis cases (**Figure 3B**). Thirty-three patients were correctly and 14 out of 47 patients were incorrectly diagnosed according to the IL-18 cut-off at < 1892.00 pg/mL for abdominal sepsis.

A receiver operating characteristic (ROC) curve for IL-18, showing an area under the curve (AUC) of 0.682 (95% CI: 0.526 to 0.839), indicates its discriminative ability (**Figure 3C**). The statistical significance is confirmed with a p-value of 0.034. This analysis underscores the potential of IL-18 as a biomarker for identifying abdominal sepsis (**Figure 3**).

### 3.6 Correlation analyses of IL-18 with clinical and inflammatory markers

The correlation analysis between IL-18 levels and a range of clinical and laboratory parameters reveals several significant associations, suggesting IL-18’s potential role as a biomarker for outcomes in ICU settings. Specifically, hematocrit levels at ICU admission were found to have a modest yet significant inverse correlation with IL-18 levels (r = −0.302, p=0.041, **Table 5**). Similarly, calcium levels, measured both at ICU admission and on d0, demonstrated moderate negative correlations with IL-18 (r = -0.348, p = 0.018, and r = -0.311, p = 0.038, respectively), suggesting a potential relationship between IL-18 levels and altered calcium homeostasis in severe cases.

**Table 5:** Correlation analyses of Interleukin-18 (IL-18) with physiological, laboratory, and clinical parameters in patients with abdominal and non-abdominal (non-abd.) sepsis at the admission to the intensive care unit (ICU adm.) or at the day of sepsis diagnosis (d0).

Correlation analyses (IL-18)	Spearman r	p value
Hematocrite (%) ICU adm.	-0.302	0.041
Ca ICU adm.	-0.348	0.018
Ca d0	-0.311	0.038
INR ICU adm.	0.379	0.010
PTT (sec.) ICU adm.	0.351	0.017
PTT (sec.) d0	0.339	0.023
GLDH (U / L) ICU adm.	0.461	0.002
GLDH (U / L) d0	0.445	0.002
GGT (U / L) d0	0.337	0.041
Troponin T ICU adm.	0.700	0.016
SOFA score ICU adm.	0.389	0.015
SOFA d0	0.376	0.017
Length of hospital stay prior ICU (days)	0.391	0.007
Length of hospital stay (days)	0.412	0.004
Mortality	0.375	0.010
MCP-1	0.525	<0.001
IL-6	0.339	0.021
IL-10	0.460	0.001

Ca, Calcium; GGT, Gamma-Glutamyl Transferase; GLDH, Glutamate Dehydrogenase; IL, Interleukin; INR, International Normalized Ratio; MCP-1, Monocyte Chemoattractant Protein-1; PTT, Partial Thromboplastin Time; SOFA, Sequential Organ Failure Assessment; Troponin T, Cardiac Troponin T. Significant correlations are highlighted with p-values, where p < 0.05 indicates a statistically significant association. The Spearman correlation coefficient (r) and p-values for various hematologic and biochemical markers, clinical scores, length of hospital stay, and inflammatory cytokines are shown.

Coagulation parameters, including INR and PTT, exhibited positive correlations with IL-18. Specifically, INR at ICU admission was positively correlated (r = 0.379, p = 0.010), as were PTT measurements at ICU admission (r = 0.351, p = 0.017) and on d0 (r = 0.339, p = 0.023), potentially indicating a link between elevated IL-18 and coagulopathy or prolonged clotting times in critically ill patients.

Transaminase levels showed particularly strong positive correlations with IL-18, with GLDH levels at ICU admission (r = 0.461, p = 0.002) and d0 (r = 0.445, p = 0.002, **Table 5**) being significant. Additionally, GGT levels on d0 also positively correlated with IL-18 (r = 0.337, p = 0.041), highlighting a possible association between IL-18 and liver function or injury markers.

Troponin T, a key cardiac biomarker, presented one of the most substantial correlations with IL-18 at ICU admission (r = 0.700, p = 0.016, **Table 5**), suggesting a potential link between IL-18 and myocardial stress or damage in the critically ill cohort.

Furthermore, SOFA scores, both at ICU admission (r = 0.389, p = 0.015 and on d0 (r = 0.376, p = 0.017, **Table 5**), were positively correlated with IL-18 levels, indicating that higher IL-18 may be associated with greater illness severity as measured by this standard scoring system.

Hospitalization metrics also correlated significantly with IL-18 levels; both the length of total hospital stay prior to ICU admission (r = 0.391, p = 0.007) and total length of hospital stay (r = 0.412, p = 0.004, **Table 5**) were positively associated with IL-18. This trend may reflect a link between elevated IL-18 and prolonged recovery or severity of illness. Additionally, mortality was positively correlated with IL-18 (r = 0.375, p = 0.010, **Table 5**), further suggesting IL-18’s potential utility as a prognostic marker for adverse outcomes.

**Figure 4:**
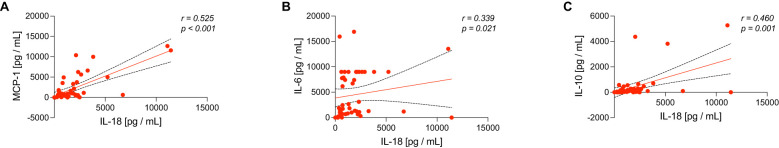
Scatter plots demonstrate correlations between interleukin-18 (IL-18) levels with **(A)** MCP-1, **(B)** IL-6, and **(C)** IL-10 levels in sepsis patients. Each plot includes a linear regression line representing the relationship between IL-18 and the respective cytokine, with Spearman correlation coefficients (r) and significance values (p) provided.

Lastly, IL-18 levels correlated with a spectrum of cytokines and chemokines involved in inflammation and immune response, including MCP-1, IL-6, and IL-10 (**Table 5**). In **Figure 4**, correlations between IL-18 levels and inflammatory markers that have shown clearly detectable levels above the minimal expression of the detection array are shown, highlighting relationships with MCP-1, IL-6, and IL-10 (p < 0.05, **Figure 4** and **Table 5**). **Figure 4A** shows a positive correlation between IL-18 and MCP-1 levels (r = 0.525, p < 0.001), indicating a notable association. **Figure 4B** presents a positive correlation between IL-18 and IL-6 with a moderate but significant correlation (r = 0.339, p = 0.021). Also, a positive correlation between IL-18 and IL-10 with a strong correlation (r = 0.460, p = 0.0.001) is highlighted in **Figure 4C**. These findings suggest that IL-18 may play a central role in the inflammatory process, correlating with multiple cytokines that are critical in both pro-inflammatory and regulatory pathways.

## 4 Discussion

Abdominal infection is a significant clinical problem, representing the second most common infection site in ICU patients (26). The diverse clinical presentations and lack of specific biomarkers make diagnosis particularly challenging and complicate the timely decision-making required for potentially early life-saving surgical infection source control.

In this mixed retrospective clinical study, we analyzed two patient groups: one with abdominal sepsis and another with sepsis from other origins. Our aim was to assess whether IL-18 could serve as a biomarker to differentiate these subtypes. Interestingly, we observed significantly higher IL-18 levels in patients with non-abdominal sepsis. While both groups were comparable in terms of age, comorbidities, and initial clinical status, the abdominal sepsis group had a length significantly shorter hospital stay prior to sepsis diagnosis, suggesting a more aggressive disease course leading to earlier ICU admission. Despite a higher rate of emergency surgical interventions in abdominal sepsis group—reflecting the need for surgical source control—SOFA and APACHE II scores at diagnosis, ICU length of stay, total hospitalization, and in-hospital mortality rates were similar between the groups. Similarly, both groups exhibited similar hemodynamic parameters, while hemoglobin and hematocrit levels in abdominal sepsis were significantly higher, likely due to pre-existing dehydration from intra-abdominal pathology. Standard inflammatory markers such as leukocyte count, PCT, and CRP, were elevated in both groups without significant distinction-indicating that these are rather not suitable to differ the sepsis subtypes. The data from respiratory parameters is limited by the higher proportion of intubated patients in the abdominal sepsis group, both at ICU admission and at the day of sepsis diagnosis.

Our primary objective was to evaluate IL-18 as a diagnostic biomarker for differentiating abdominal from non-abdominal sepsis. Surprisingly, IL-18 levels were significantly higher in patients with non-abdominal sepsis, a result that contrasts the study by Mierzchala-Pasierb et al., who reported elevated IL-18 levels in abdominal sepsis compared to pulmonary sepsis (20). This discrepancy may stem from methodological differences, including their limited sample size (n=9 for abdominal sepsis, n=8 for pulmonary sepsis) and the notably higher SOFA scores in the abdominal sepsis group. Furthermore, while their comparison focused solely on abdominal versus pulmonary sources, our non-abdominal cohort was more diverse, encompassing pneumonia, urosepsis, and empyema. IL-18 levels in our cohort correlated with several key inflammatory mediators, including MCP-1, IL-6, and IL-10, suggesting its broader immunologic role in the septic response. Several pro-inflammatory cytokines, such as MCP-1 and IL-6, were significantly elevated in both sepsis groups compared to healthy controls. MCP-1, expressed by various cells, including fibroblasts, endothelial cells, and smooth muscle cells, plays a pivotal role in the immune response to sepsis (27). It correlates with SOFA scores (28) and 28-days mortality (29). IL-6, a multifunctional cytokine, initiates acute-phase reactions, enhances leukocyte recruitment, and increased vascular permeability, all of which can contribute to tissue damage and organ dysfunction in septic shock (30). IL-6 also promotes the production of IL-10, an anti-inflammatory cytokine that helps control excessive immune activation (30). While IL-6 is a sensitive diagnostic biomarker for sepsis (85.0% sensitivity; and 62.0% specificity) (31) and correlates with sepsis-related mortality (32), it lacks specificity for abdominal sepsis. Therefore, while both MCP-1 and IL-6 hold prognostic value, they are not suitable for distinguishing abdominal sepsis.

IL-18`s behavior in different sepsis remains complex and may depend on microbial etiology. Previous studies have reported conflicting data: Mierzchala-Pasierb et al. found no significant IL-18 differences between Gram-positive and Gram-negative bacterial infections, while Oberholzer et al. noted elevated IL-18 in Gram-positive infections (20, 33). These inconsistencies underscore the heterogeneity of IL-18 responses in sepsis and suggest a need for larger, more stratified studies. From a biological standpoint, IL-18 is a highly plausible candidate as a sepsis biomarker. As a proinflammatory cytokine in the IL-1 family, it plays a critical role in innate immunity and stimulates interferon-gamma release (20–22). It also activates the NF-κB pathway, promoting the expression of adhesion molecules, chemokines, and Fas ligand, all central to the inflammatory cascade in sepsis (22). In experimental models, IL-18 has been linked to gut barrier dysruption, increased bacteremia, and sepsis-related mortality (34). In fact, simultaneous blockade of IL-1 and IL-18 has been shown to confer full protection in murine models of sepsis induced by LPS or cecal ligation and puncture (35) (36). Clinically, IL-18 correlates with critical severity indicators such as age, temperature, respiratory rate, and CRP (37). Elevated IL-18 levels have been observed in both adult and neonatal sepsis, especially in non-survivors (34). Compared to traditional markers like CRP, PCT, and white blood cell count (WBC), IL-18 has demonstrated better diagnostic discrimination for sepsis and septic shock (20). Given the limited sensitivity and specificity of leukocytosis and limited specificity of existing markers like CRP and PCT—both of which can be elevated in trauma and postoperative states (16)—IL-18 may offer a more precise means of early diagnosis and should be further elaborated in larger studies. Similarly, lactate though assessing perfusion deficits, remains non-specific for infection source (18), and although IL-6 is widely used and linked to sepsis severity, it also lacks specificity for distinguishing abdominal from non-abdominal sepsis (19).

IL-18`s established role in mucosal immunity, particularly in inflammatory bowel disease (22–24), further supports its potential relevance in abdominal infections. However, its prognostic utility remains controversial. While Mierzchala-Pasierb et al. and Esquerdo et al., found no significant difference between survivors and non-survivors (20, 38), others—such as Eidt et al.—reported a positive association between IL-18 and mortality (39). Our data support the latter, reinforcing the potential of IL-18 not only as a diagnostic marker but also as a prognostic tool in critically ill patients. Finally, the broader clinical relevance of IL-18 extends beyond sepsis. It has been implicated in various inflammatory and autoimmune diseases, including rheumatoid arthritis, psoriasis, systemic lupus erythematosus, adult-onset Still’s disease, and dermatomyositis (40), which further highlights its broader immunological role.

In conclusion, our findings add to the growing body of evidence supporting IL-18 as a promising biomarker in sepsis. Despite conflicting reports in the field, its strong biological plausibility, clinical correlations, and potential for infection source differentiation justify further investigation, particularly in distinguishing abdominal from non-abdominal sepsis.

## 5 Limitations

The study has several limitations that should be acknowledged. First, the retrospective design may introduce selection bias and limit the generalizability of the results to broader ICU populations or different healthcare systems. Second, the overall sample size was relatively small (n=47), which reduces statistical power and may increase the likelihood of some errors like type II, particularly in subgroup comparisons. Third, the non-abdominal group was heterogenous, including infections such as pneumonia, urosepsis, and emphyema, which may have contributed to variability in inflammatory responses and limited the ability to detect source-specific cytokine patterns. Of limiting importance is the younger age in the group of healthy controls which should be matched in future studies. Furthermore, the study relies on single-time-point measurements of cytokines (on the day of sepsis diagnosis), which may not capture the dynamic changes in respiratory and inflammatory parameters over the course of the disease. This limitation is especially relevant for cytokines such as IL-18, where fluctuations over time could provide additional diagnostic and prognostic value. While IL-18 showed moderate diagnostic accuracy in our study (AUC 0.68), its sensitivity and specificity were rather insufficient to be used as a sole biomarker for abdominal sepsis. Additional confounding factors were not fully addressed. For instance, differences in ventilatory support strategies–evidenced by the unequal distribution of ventilated patients and higher PEEP in abdominal sepsis patients–as well as the unaccounted effects of sedation and catecholamine administration, could influence the observed hemodynamic parameters. Moreover, other factors such as prior antibiotic therapy, variability in the timing of surgical interventions, and other clinical management practices were not standardized across patients and may have affected cytokine levels and clinical outcomes. Also, the study was not adjusted for those potential confounders (e.g., timing of antibiotic administration, immunosuppression, comorbidities), which limits the validity of the cytokine comparisons. Although we evaluated several commonly used biomarkers, pathogen-specific analyses (e.g., Gram-positive versus Gram-negative infections) were not performed. Given that microbial etiology can influence immune responses, this represents a missed opportunity for further stratification. Finally, the findings were not validated in an external cohort, and larger, prospective, and multicenter studies with longitudinal biomarker profiling and standardized treatment protocols are needed to confirm the diagnostic and prognostic utility of IL-18 and other cytokines in sepsis.

## 6 Conclusions

This study evaluated the clinical profiles and immunological responses of patients with abdominal versus non-abdominal sepsis, with a particular focus on the diagnostic utility of IL-18. While standard clinical parameters and inflammatory markers such as CRP, PCT, and IL-6 were elevated in both groups, they lacked the specificity to distinguish sepsis origin. IL-18 levels were significantly higher in non-abdominal sepsis and correlated with other key cytokines, including IL-6, IL-10, and MCP-1, suggesting a broader role in systemic inflammation. Despite showing moderate diagnostic accuracy (AUC 0.68), IL-18 alone may not be sufficient as a standalone biomarker for identifying abdominal sepsis. However, its biological plausibility, correlation with disease severity, and ability to reflect distinct immunologic profiles support its continued investigation. These findings underscore the need for a multimodal diagnostic approach combining clinical evaluation with a panel of biomarkers. Given the limitations of this retrospective study—including small sample size, heterogeneous infection sources, and single time-point cytokine measurements—larger prospective studies with longitudinal assessments are warranted to validate IL-18’s diagnostic and prognostic value in sepsis, particularly in distinguishing abdominal from non-abdominal infection sources.

## Data Availability

The original contributions presented in the study are included in the article/supplementary material. Further inquiries can be directed to the corresponding author.
